# The impact of demographic and clinical characteristics on the trajectories of health-related quality of life among patients with Fabry disease

**DOI:** 10.1186/s13023-021-02066-y

**Published:** 2021-10-12

**Authors:** Solrun Sigurdardottir, Birgitte Bjerkely, Trond G. Jenssen, Per Mathisen, Charlotte von der Lippe, Kristin Ørstavik, Ketil Heimdal, Dag Olav Dahle, Mina Susanne Weedon-Fekjær, Olga Solberg, Hege K. Pihlstrøm

**Affiliations:** 1 Centre for Rare Disorders, Oslo University Hospital, Postboks 4950 Nydalen, 0424 Oslo, Norway; 2 Department of Surgery, Inflammation Medicine and Transplantation, Section of Nephrology, Oslo University Hospital, Rikshospitalet HF, Oslo, Norway; 3 Institute of Clinical Medicine, Faculty of Medicine, University of Oslo, Oslo, Norway; 4 Department of Cardiology, Oslo University Hospital, Rikshospitalet HF, Oslo, Norway; 5 Department of Neurology, Section for Rare Neuromuscular Disorders, Oslo University Hospital, Rikshospitalet HF, Oslo, Norway; 6 Department of Medical Genetics, Oslo University Hospital, Rikshospitalet HF, Oslo, Norway

**Keywords:** Quality of life, HRQOL, SF-36, Longitudinal studies, Fabry disease, ERT, Kidney disease, Neuropathic pain

## Abstract

**Background:**

Fabry disease (FD) is an X-linked lysosomal storage disorder characterized by multiorgan dysfunction. Since individuals with FD usually experience progressive clinical disease manifestations, their health-related quality of life (HRQOL) is expected to change over time. However, there is limited longitudinal research examining HRQOL outcomes in individuals with FD. We aimed to: assess longitudinal outcomes in HRQOL in adults with FD; examine the physical- and mental HRQOL trajectories at the initial registration (baseline), 3–5 year, and 7–13 year follow-ups; and evaluate the possible associations of age, sex and medical complications with the physical- and mental HRQOL trajectories.

**Methods:**

Forty-three individuals with FD (53% female) who were aged 18 to 81 years at baseline attended clinical follow-up visits between 2006 and 2020. Medical records were extracted retrospectively. Demographics and the 36-item Short-Form Health Survey (SF-36) were recorded at scheduled visits, except for the last data collection which was prospectively obtained in 2020. The physical (PCS) and mental (MCS) composite scores (SF-36) were chosen as outcome measures.

**Results:**

The eight SF-36 domain scores were stable over a span of 13 years, and only physical- and social functioning domains worsened clinically over this follow-up period. Mean baseline SF-36 domain scores were all significantly lower (decreased HRQOL) in the FD sample compared with Norwegian population norms. Two hierarchical linear models were run to examine whether demographics and medical complications (measured at the last clinical visit) predicted physical and mental HRQOL trajectories. Age above 47 years (*p* < 0.001), male sex (*p* = 0.027), small fibre neuropathy (*p* < 0.001), renal dysfunction (*p* < 0.001), and cerebrovascular events (*p* = 0.003) were associated with lower HRQOL over time. No significant interactions were found between the time of follow up and the abovementioned predictors of HRQOL.

**Conclusions:**

Overall HRQOL trajectories remained stable between baseline, 3–5 year, and 7–13 year follow-ups, with the majority of individuals reporting decreased physical and mental HRQOL. Medical complications in combination with older age and male sex are important predictors of lower HRQOL in FD. Awareness of this relationship is valuable both for health care providers and for patients. The findings provide indicators that can guide treatment decisions to improve physical and mental HRQOL outcomes.

**Supplementary Information:**

The online version contains supplementary material available at 10.1186/s13023-021-02066-y.

## Introduction

Fabry disease (FD; OMIM 301500) is a life-threatening X-linked lysosomal storage disorder caused by a pathogenic variant in the GLA-gene resulting in deficiency of the enzyme α-galactosidase A (α-Gal-A) [1]. Subsequent accumulation of globotriaosylceramide causes progressive tissue damage that leads to cardiovascular [2, 3], renal [2, 4] and cerebrovascular dysfunction [5–7]. Neuropathic pain is typical, especially in male patients, often starting in early childhood and triggered by fever, fatigue or stress [8, 9]. FD is divided into classic early-onset disease (severe course with no residual enzyme activity) or nonclassical later-onset disease (milder course with residual enzyme activity) [10, 11]. Disease-specific treatment involves enzyme replacement therapy with regular intravenous infusions of α-Gal-A or α-Gal-B [12, 13]. The classic phenotype occurs in 1:40,000 males [14]. Females are also affected with FD manifestations ranging from mild to severely symptomatic, usually with a later age of onset [15, 16]. The multifaceted impact of the medical complications associated with FD may have long-lasting consequences for the individual’s physical and mental functioning. Ongoing difficulties with pain, fatigue, anxiety, depression, and employment are reported in adult life [17–19]. The importance of including both medical indicators and health-related quality of life (HRQOL) measures in clinical FD follow-ups has been recognized [10, 20, 21]. HRQOL is a multidimensional concept referring to “the subjective evaluation of the impact of the health status in domains related to physical, mental, emotional, and social functioning” [22].

Studies report that FD may affect all aspects of life, resulting in decreased HRQOL in both males and females compared to healthy individuals [11, 23–25]. The factors that are reported to influence HRQOL with FD are disease severity [11, 26], phenotype [11], cardiovascular complications [20, 27], stroke or transient ischaemic attacks [27], kidney disease [20, 27], gastrointestinal pain [28, 29], neuropathic pain [11, 20] and small fibre neuropathy [30]. Some studies have found enzyme replacement therapy (ERT) to have a positive effect on HRQOL [31–33] while others have reported no relationship [23, 34, 35]. Men and women with FD may experience reduced HRQOL, but generally younger males (18–25 years of age) report lower HRQOL than younger females [21]. Older individuals (> 50 years) with classical FD have lower HRQOL than younger individuals [11, 15, 21].

Investigations of longitudinal changes in quality of life in populations with chronic and rare disorders are generally scarce [22]. Longitudinal studies in which the same variables are measured over time for the same sample of FD patients are needed. This research is a retrospective longitudinal study including data on individuals with FD collected over a decade. The aims were to: assess longitudinal outcomes in HRQOL in adults with FD across ages and sex using eight domains of the Medical Outcomes Scale 36-item Short-Form Health Survey (SF-36); examine the physical- and mental HRQOL (SF-36) trajectories in adults with FD at baseline, 3–5 year, and 7–13 year follow-ups; and evaluate the associations of demographic and medical complications with the physical- and mental HRQOL trajectories.

## Methods

### Study design and patient population

This study included individuals with FD who were being followed in a clinical program at Oslo University Hospital, Norway, between 2006 and 2020. Inclusion criteria were a likely pathogenic or pathogenic mutation in the α-GLA gene and age ≥ 18 years. Data from medical records covering the period 2006–2019 were extracted retrospectively. A total of 46 persons with FD (19 males and 27 females) with an average age of 49.4 years who were followed up between January 2006 and June 2020 were invited to participate in the study. They received written information about the study by mail (March 2020) or in conjunction with their clinical follow-up assessments in the period April to August 2020. Ten persons declined to participate. There were no significant differences between participants (n = 36) and nonparticipants (n = 10) regarding age (*p* = 0.827) or sex (*p* = 0.412). Participants completed questionnaires that assessed quality of life, pain, fatigue, and mental health. The Norwegian Regional Committee for Medical Research Ethics in southeastern Norway gave permission to include data for deceased individuals. The final sample included 43 persons (20 males and 23 females), including those who died (n = 7) in the 2006–2019 period.

### Clinical and laboratory measures

In the period 2006–2019, HRQOL was assessed using the SF-36 at clinical follow-ups at the hospital. The SF-36 data from 2020 were collected by post in sealed envelopes and included 36 individuals. A total of 148 SF-36 questionnaires were completed by participants and deceased individuals from baseline to 13-year follow-up. For this paper, we chose to include SF-36 scores representing three distinct time periods. The 3–5 and 7–13 year timeframes were selected in order to include maximum amount of longitudinal data collection. There were 43 (100%), 30 (86%) and 16 (39%) patient SF-36 assessments at baseline (1st clinical visit), between 3–5 year follow-ups, and 7–13 year follow-ups, respectively. The last SF-36 data obtained in the 3–5 year and 7–13 year follow-ups were included. Fourteen patients had more than three SF-36 assessments and these data were excluded (59 data points). Additional file 1: Bar chart 1 shows the total number of SF-36 records at each year of follow-up from baseline to 13 years. These are grouped by SF-36 or SF-36 not included at baseline (1st clinical visit), between 3–5 year follow-ups, and 7–13 year follow-ups.

Routine clinical and laboratory data were collected by physicians and nurses. Cardiac, kidney, small fibre function, and cerebral parameters were measured repeatedly over several years using echocardiography, electrocardiogram, laboratory data, quantitative sensory testing and brain magnetic resonance imaging (MRI at baseline and then every two years). These medical parameters were collected at the first visit (2002—2020) and the last clinical visit (2009—2020). Clinical data at the first visit had more missing values than at the last visit; therefore, the last clinical visit parameters were chosen as predictors of HRQOL. These parameters were collected at least 4 months before the study’s inclusion, which started in March 2020. In four cases, the last clinical visit data were collected at the same point of time as the SF-36 outcome data in 2020.

Of note, the phenotypical classification of classical or nonclassical FD was not applied in this study. Instead, participants (males) were categorized on the basis of α-Gal-A enzyme activity using the cut off < 2%.

*Cardiac parameter.* Changes in the myocardium were quantified by measurements of septal wall thickness and cardiac ejection fraction (EF ≤ 50%). Left ventricular hypertrophy was defined as a septal wall thickness > 11.5 mm. Previous or persistent arrhythmia, angina pectoris, myocardial infarction, coronary stent implantation, and coronary artery bypass surgery were defined as cardiovascular events. Pacemakers and implantable cardioverter defibrillators (ICDs) were registered.

*Small fibre neuropathy* was assessed by quantitative sensory testing. Heat detection thresholds, cold detection thresholds, heat pain detection thresholds, and cold pain detection thresholds were determined using a computerized Thermotest® (Somedic AB, Sweden). Heat- and cold detection thresholds were calculated as the average of five consecutive temperature recordings. These thresholds were determined at the thenar eminence of one hand, at the lateral aspect of one thigh, at the lateral aspect of both legs approximately 15 cm below the knee level, and at the dorsum of both feet. Thresholds were compared to healthy controls tested for quantitative sensory testing in our lab [36]. Findings of increased thresholds for either heat- or cold detection thresholds or both at the dorsum of the feet indicated a small fibre neuropathy.

*The measured glomerular filtration rate* (mGFR) is the gold standard for assessing renal function in adults with FD [37], and we used the fractional reduction in plasma activity of technetium (99mTc-DTPA) at four time points after intravenous injection. Renal replacement therapy i.e., haemodialysis or kidney transplantation, was registered (yes/no).

*Cerebral parameter*. Our definition of a cerebrovascular event included a history of clinical stroke or transitory cerebral ischaemic event or evidence of a previous ischaemic event on brain MRI scan.

*Treatment.* Enzyme replacement therapy (ERT) was agalsidase alfa (Replagal) or agalsidase beta (Fabrazyme) given every two weeks by intravenous infusion. Another pharmacologic treatment of FD was chaperone migalastat. In total, 36 participants were receiving Fabry-specific treatment in 2020.

### Self-reported HRQOL outcomes

*The Short Form (*SF-36v2®*) Health Survey* is a 36-item self-evaluation instrument designed to measure the impact of disease on activities of daily living and quality of life [38]. The SF-36 is a generic measure and can be used across age (18 years and older), diseases and treatment groups, including FD [23]. The SF-36 is a patient reported outcome measure that allows comparison of quality of life with other chronic diseases and may capture changes in HRQOL or symptoms over time. However, SF-36 is not a disease specific measure and this may limit its use in people with chronic diseases since it might not capture the typical symptoms and burdens associated with these conditions, which can have a significant impact on HRQOL.

The SF-36 consists of eight health domains: (PF) Physical Functioning; (RP) Role-Physical; (BP) Bodily Pain; (GH) General Health; (VT) Vitality; (SF) Social Functioning; (RE) Role-Emotional; and (MH) Mental Health. A licence from Quality Metric was used for scoring (Licence Agreement QM049911). Domain raw scores were transformed into a scale score with a range of 0–100 (worst to best). According to data from studies in normal populations, a 10-year change of > 5 points from baseline was considered clinically significant for each of the eight SF-36 domains [38, 39]. Age- and sex-matched normative data from the Norwegian population were used for comparison [40]. The eight domains were weighted and summarized into physical component summary (PCS) scores and mental component summary (MCS) scores. The PCS and MCS are expressed as standardized T-scores (mean value of 50, SD ± 10). Higher scores indicate better HRQOL.

### Ethical statement

All protocols and methods were approved by the Norwegian Regional Committee for Medical Research Ethics in southeastern Norway (number 31513) and the Data Protection Officer at Oslo University Hospital (number 20/01134). Information about the study and questionnaires was distributed by mail, and written informed consent was collected from all participants. FD-related information was retrospectively collected from medical records after consent had been received.

### Statistical analysis

Statistical analyses were performed using IBM SPSS 25 Statistics. Means and standard deviations (SDs) are presented for continuous normally distributed data; medians and interquartile ranges (IRs) are presented for non-normally distributed data; and frequencies are presented for categorical data. *T-*tests, Mann–Whitney U tests and *X*^*2*^ tests were used to examine differences in age and medical variables between males and females. The SF-36 component scores (PCS, MCS) showed a normal distribution using the Kolmogorov–Smirnov test. Hierarchical linear modelling (HLM) analysis was used to examine potential predictors for the SF-36 component scores (PCS, MCS). The SF-36 scores were used as dependent variables with three time points coded as 0 (baseline), 4 (3–5 years), and 10 (7–13 years). The following covariates were included in the model: sex, age at baseline, cardiac EF ≤ 50% (yes/no), small fibre neuropathy (yes/no), mGFR (ml/min), and a history of cerebral vascular disease (yes/no). Covariate data were entered simultaneously as fixed effects. Age and mGFR were centralized with the total sample mean values before being entered into the HLM. The model handled missing data (PCS, MCS) at the follow-ups through maximum likelihood estimation, thus retaining all 43 participants. Statistically significant fixed effects on the SF-36 scores were then graphed across each of the time points. The main effect would indicate that the SF-36 scores over time vary as a function of the predictor variable. Uncertainty in the PCS and MCS estimates was evaluated using a bootstrap with 1000 replications. The statistical significance was set at *p* < 0.05.

## Results

### Sample characteristics

Descriptive statistics for demographics and medical characteristics at the last clinical visit are presented in Table 1 for the total sample (n = 43) and for men and women. Fifty-three percent of the participants were females, and the mean (SD) age at baseline was 47.0 ± 15.3 years (range 18–81). The most frequent clinical manifestations were left ventricular hypertrophy (59%), cornea verticillata (56%), reduced hearing (50%), small fibre neuropathy (49%), pre-cerebral vessel arteriosclerosis (43%) and a history of a cerebrovascular event (35%). A total of 12 males (60%) had α-GAL-A enzyme activity < 2%. More male (75%) than female (26%) subjects experienced small fibre neuropathy (*p* < 0.001). Similarly, only men (26%) received Renal replacement therapy. Of the participants who were alive in 2020, all males received either ERT (n = 11) or chaperone (n = 5), while 50% of females received either ERT (n = 9) or chaperone (n = 1) in 2020. The mean duration of Fabry-specific treatment was 9.6 years (SD 7.0) for males and 5.2 years (SD 5.6) for females (*p* = 0.09).

**Table 1:** Demographics and clinical characteristics of the study sample

Demographics	Total	Men	Women	*p*
	n = 43	n = 20 (47%)	n = 23 (53%)	
Age in 2020 (range 21–78) (n = 36)	49.8 (15.1)	50.2 (13.1)	48.2 (16.8)	.701
Age at first clinical visit (range 18–81)	47.0 (15.3)	44.4 (8.8)	49.2 (19.2)	.313
*Medical variables last clinical visit*				
α-GAL-A enzyme activity < 2%	12 (28%)	12 (60%)	0 (0%)	
Cornea verticillata (n = 41)	23 (56%)	8 (44%)	15 (65%)	.183
Left ventricular hypertrophy (n = 41)	24 (59%)	12 (63%)	12 (55%)	.577
Cardiac ejection fraction (EF ≤ 50%)	10 (23%)	6 (30%)	4 (17%)	.329
Pre-cerebral vessel arteriosclerosis (n = 40)	17 (43%)	10 (56%)	7 (32%)	.131
Pacemaker/ICD implant	8 (19%)	5 (25%)	3 (13%)	.315
Clinical history of cerebrovascular event^a^	15 (35%)	8 (40%)	7 (30%)	.512
Small fibre neuropathy	21 (49%)	15 (75%)	6 (26%)	.**001**
Measured GFR (ml/min)	69.0 (26.5)	62.8 (30.1)	74.4 (22.6)	.154
Renal Replacement Therapy (tx/dialysis)	5 (12%)	5 (26%)	0 (0%)	**.012**
Reduced hearing/tinnitus	21 (50%)	11 (55%)	10 (46%)	.537
Lyso-GB3 (ng/ml) (n = 36)	5.0 (8.2)	7.3 (27.3)	4.9 (6.3)	.188
Currently on ERT/chaperone (n = 36)	26 (72%)	16 (100%)	10 (50%)	**.001**
*Medical conditions up to last clinical visit*				
Gastrointestinal disease (acute or chronic)	14 (33%)	4 (20%)	10 (44%)	.101
Diabetes mellitus	2 (5%)	1 (5%)	1 (4%)	.919
History of cancer	9 (21%)	3 (15%)	6 (26%)	.373
Chronic obstructive pulmonary disease	12 (28%)	7 (35%)	5 (22%)	.334
Coronary artery stenoses (by angiography)	4 (10%)	3 (15%)	1 (5%)	.249

The significance of bold is p < 0.05

Values are mean (± SD) or n (%) except for Lyso-GB3 with values median (IR)

Gender differences using t-test or χ^2^-test, as appropriate. Abbreviations: α-GAL-A, enzyme α-galactosidase A; ERT, enzyme replacement therapy; GFR, Glomerular filtration rate; ICD, implantable cardioverter-defibrillator

^a^Clinical stroke /transitory ischaemic event, or evidence of a previous ischemic event on MRI brain scans

### The SF-36 domain score changes over time

**Fig. 1:**
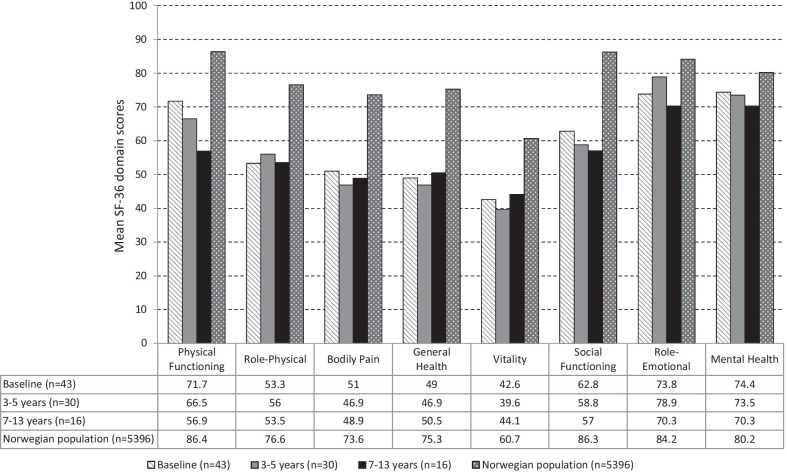
SF-36 domain scores for the FD sample at each follow-up compared to the Norwegian population. *Note.* Mean scores for males and females for the SF-36 domains are presented in Additional files 2 and 3, respectively

The SF-36 domain scores remained unchanged across ages and sex from baseline up to the 7–13 year follow-up. Figure 1 illustrates the mean SF-36 domain scores at each selected time frame in comparison with normative data in a Norwegian cross sectional study [40]. Clinically significant negative changes (mean difference > 5 points) [39] occurred in two domains only, physical functioning and social functioning, between baseline and 7–13 year follow-ups. The role emotional domain showed an improvement up to 3–5 years, followed by a decrease between 3–5 years and 7–13 years. The Vitality domain showed the lowest mean score compared to other domains. The FD study sample showed significantly lower domain scores across all eight domains at baseline (PF, RP, BP, GH, VT, and SF *p* < 0.001; RE and MH *p* < 0.05) compared to values from a Norwegian general population cohort [40]. Comparisons between males and females with FD and Norwegian general population SF-36 scores for both sexes [41] are presented in Additional file 2: Fig. S1A—Males and Additional file 3: Fig. S1B—Females.

### Predictors of HRQOL trajectories

*Unconditional Models.* The PCS model showed a significant estimated subject variance of 122.63 (Wald Z = 2.62, *p* < 0.01) and a significant estimated residual variance of 32.66 (Wald Z = 4.79, *p* < 0.001). The MCS model showed a nonsignificant estimated subject variance of 24.59 (Wald Z = 1.74, *p* = 0.08) and a significant estimated residual variance of 67.41 (Wald Z = 4.90, *p* < 0.001). These analyses suggest that there was a sufficiently large score variance within subjects to proceed with the HLM. A spaghetti plot of 30 participants demonstrated interindividual variability in MCS scores between baseline and the 3–5 year follow-up (see Additional file 4). Unconditional linear, quadratic and cubic models were then analysed without the covariates for PCS and MCS scores over time (see Additional file 5).

*Full models.* The two HLM models fitted for the PCS and MCS scores were constructed using 129 observations from 43 participants. The full HLM models examined whether linear HRQOL trajectories over time could be predicted by sex, age at baseline and medical complications assessed at the last clinical visit. Bivariate correlations among predictors of HRQOL and the PCS and MCS baseline scores are presented for reference (see Table 2).

**Table 2:** Bivariate correlations between demographics, medical conditions and HRQOL trajectories

Variables	Physical HRQOL	Mental HRQOL	Sex	Age	EF	Neuro-pathy	Higher GFR	Cerebro-vascular
MCS baseline	.07	–						
Sex	.23	.05	–					
Age at diagnosis	− .11	.01	.16	–				
Cardiac ejection fraction (EF ≤ 50%)	.35**	− .12	.15	− .48**	–			
Small fibre neuropathy	.40**	.09	.49**	− .25	.45**	–		
Higher GFR (ml/min)	.37**	.06	.22	− .43**	.36*	.18	–	
History of cerebrovascular events	.24*	.19	.10	− .20	.18	.07	.32*	–

*p < 0.05; **p < 0.01

HRQOL = Health-related quality of life (SF-36); GFR = Glomerular filtration rate

**Fig. 2:**
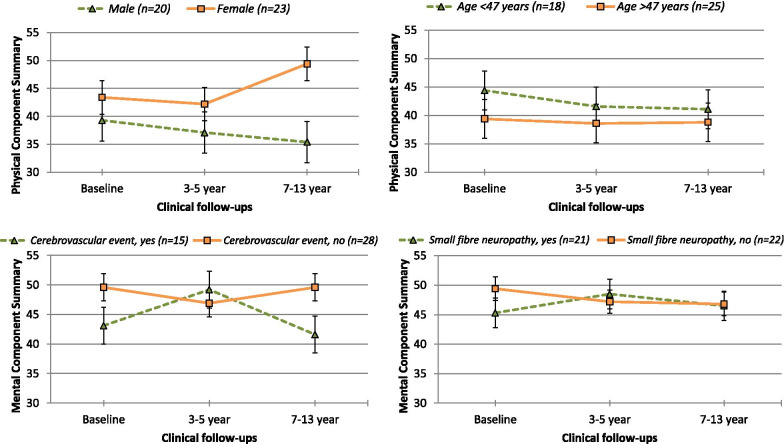
Significant predictors for the Physical Component Summary and Mental Component Summary HRQOL trajectories (SF-36). *Note*. Scores are presented by T-scores (mean 50, standard error)

The covariates in the models (PCS and MCS) are presented in Table 3, which shows their standardized coefficients (*β*) and statistically significant and nonsignificant fixed effects from the HLM, as well as 95% confidence intervals. Across the three time points, no significant changes were found in the PCS and MCS trajectories (*p* > 0.05). Figure 2 depicts the PCS trajectories of sex and age and the MCS trajectories of small fibre neuropathy (yes, no) and history of cerebrovascular events (yes, no). Female sex (*p* < 0.05) and younger age (*p* < 0.001) were significantly associated with better physical HRQOL. Male participants in particular demonstrated declines in physical HRQOL (see Fig. 2), although this group size was small (*n* = 20); thus, confidence intervals were wide for the HRQOL variable. Participants without small fibre neuropathy and those with higher mGFR values (Table 3) showed better physical HRQOL (*ps* < 0.001). There were no trends regarding the association of age and sex on mental HRQOL, but there was substantial variation among individual trajectories (see Additional file 4), as evidenced by the wide prediction intervals. Participants with EF < 50% (*p* = 0.003) and without small fibre neuropathy (*p* = 0.031) had better mental HRQOL (see Fig. 2). In each of the HRQOL models (Table 3), those without a history of cerebrovascular events had better physical- and mental HRQOL (*ps* < 0.01). There were no statistically significant interactions between time at follow-ups and the identified significant predictors for the physical and mental HRQOL trajectories (Table 3). This suggests that the slopes of the participants’ HRQOL trajectories did not change differentially over time as a function of sex, age, small fibre neuropathy, mGFR or history of cerebrovascular event.

**Table 3:** Predictors of each quality of life trajectory (SF-36) across baseline, 3–5 year and 7–13 year follow-ups

Predictor variable	Physical Component Summary (SF-36)					Mental Component Summary (SF-36)				
	*β*	S.E	*p*	95%CI Lower Bound	95%CI Upper Bound	*β*	S.E	*p*	95%CI Lower Bound	95%CI Upper Bound
Intercept	32.21	2.35	.001*	26.83	36.11	47.57	2.32	.001*	42.82	51.94
Time of assessment	− 0.05	0.24	.852	− 0.51	0.46	− 0.08	0.24	.775	− 0.51	0.40
Male sex	− 5.24	2.34	.027*	− 8.64	0.73	− 0.07	0.22	.731	− 5.95	4.80
Younger age at diagnosis	0.25	0.06	.001*	0.13	0.37	0.03	0.07	.544	− 0.10	0.17
Cardiac ejection fraction (EF ≤ 50%)	3.14	2.53	.148	− 1.66	8.19	− 6.85	2.74	.003*	− 11.89	− 0.95
Small fibre neuropathy	11.66	2.29	.001*	6.59	15.51	5.24	3.01	.031*	− 0.74	11.17
Higher GFR (ml/min)	0.21	0.04	.001*	0.12	0.28	0.03	0.04	.326	− 0.06	0.12
History of cerebrovascular events	5.12	1.99	.003*	1.65	9.34	4.51	1.96	.004*	0.70	8.16
− 2LL without interaction	636.9					642.9				
Time*Significant predictors										
Time*Gender	0.25	0.76	.771	− 1.46	1.62	–	–	–	–	–
Time*Age	− 0.01	0.03	.655	− 0.06	0.05	–	–	–	–	–
Time*Cardiac EF ≤ 50%	–	–	–	–	–	− 0.21	0.80	.796	− 1.45	1.76
Time*Small fibre neuropathy	0.43	0.69	.560	− 1.04	1.64	− 0.05	0.80	.943	− 2.00	1.28
Time*Measured GFR (ml/min)	− 0.01	0.01	.404	− 0.03	0.01	–	–	–	–	–
Time*History of cerebrovascular event	− 0.30	0.76	.713	− 1.78	1.21	0.67	0.63	.916	− 1.47	1.05
− 2LL with interaction	631.7					642.7				

* = *p* < .05

Predictor variables (age, GFR) are centered at mean. Lower scores on the measured GFR indicate worse renal function

Abbreviations: EF, ejection fraction; GFR, Glomerular filtration rate

## Discussion

To the best of our knowledge, this is the first study to present longitudinal outcomes in HRQOL in adults with FD, with observations covering more than a decade. A main finding was that the FD sample exhibited stable scores of both physical and mental HRQOL trajectories over the course of 13 years. Two HLM models were run to examine the association of age, sex and medical complications with physical and mental HRQOL trajectories. The analyses showed that sex, age, small fibre neuropathy, mGFR, cardiac ejection fraction and a history of cerebrovascular events were significant predictors of HRQOL trajectories. No significant interactions were found between the time of follow-ups and the abovementioned predictors of HRQOL.

Individuals with FD in the current study had lower SF-36 domain scores (lower HRQOL) than the general population [40]. These results are in line with a larger review [23] and original studies that found decreased HRQOL in individuals with FD [11, 20, 21, 26, 27]. Furthermore, the SF-36 domain mean scores were similar to those reported by a recent Brazilian study in FD [26] and a Dutch study in 2013 [42]. Notably, the SF-36 mean scores were generally higher in recent FD studies than scores reported in an FD study in 2002 [27]. This difference may be associated with different selection of samples with FD and that individuals included in recent studies may have milder FD phenotypes or receive better FD treatments.

In this retrospective longitudinal study, HRQOL trajectories remained relatively stable in the FD group from baseline and assessments at the 3–5 year and 7–13 year follow-ups. Since individuals with FD usually have many medical sequelae, their HRQOL is predicted to be negatively affected and expected to change over time. Other studies have shown that individuals with FD decline in HRQOL with disease progression over time [21, 23, 33]. For example, one study observed that HRQOL declined in 42% of individuals with FD over a 4-year period in a home-based ERT program [32]. In our sample of Norwegian patients with FD, negative changes over time occurred only in the domains of physical functioning and social functioning (a scale difference of ≤ 5), suggesting more difficulties in physical health, personal relationships and perhaps social difficulties. Future studies should investigate whether interventions that target physical and mental health as well as social participation may improve HRQOL over time in individuals with FD.

Age above the mean value (> 47 years) in our study was associated with decreased physical HRQOL over time, in line with past research that found that older age (> 50 years) in FD was related to lower HRQOL [11, 21]. It is possible that HRQOL scores in older individuals may reflect FD disease severity as previously reported [23, 35], rather than an association of age alone. Data reported for the Norwegian population show that older age (65–96 years) was negatively associated with all SF-36 scale scores [41]. This comparison with normative data suggests that HRQOL starts to decline at a much younger age in individuals with FD in our study than in the general population and maybe as young as 25 years of age as reported by Wilcox et al. [21]. Decreased physical HRQOL was reported by males in our study. In other studies, the relationship between age and sex was a contributing factor of lower HRQOL, with significantly lower HRQOL in untreated males with classic FD after the age of 40 [27] and in treated males with classic FD after the age of 50 years [11]. The HRQOL scores observed for females with FD at baseline in our study were lower than those of the normal female population. Physical decline at age 55 years among females with FD was reported in another study [21] and resembled the HRQOL reported for females with multiple sclerosis and rheumatoid arthritis [25].

Decreased HRQOL in our FD sample and the contribution of medical complications (heart, kidney, and cerebrovascular) to that relationship shown in the two HLM models corroborate previous studies [20, 27, 30]. For instance, our results revealed that individuals with low mGFR (worse renal function) showed decreased physical HRQOL, consistent with another study that reported estimated GFRs [20]. However, renal function did not predict mental HRQOL. An impact of mGFR on physical rather than mental HRQOL trajectories would seem more probable, as more severe renal dysfunction inevitably leads to worsening of physical function. Furthermore, the presence of small fibre neuropathy, often manifested by chronic pain in the extremities, had a negative impact on both physical and mental HRQOL. To the best of our knowledge, the use of thermal threshold investigations to predict HRQOL in FD has not been previously described. Although no linear relationship has been established between subjective pain and small fibre function in FD [43], neuropathic pain is regarded as one of the most challenging aspects of FD [11, 44]. Other studies investigating HRQOL in FD found that subjective pain affected mood and enjoyment of life [24], as well as HRQOL [20].

One-third of the patients with FD in our study had a history of at least one cerebrovascular event, which was associated with lower scores on both physical and mental HRQOL over time. It is well known that persons with FD may have cognitive deficits after cerebrovascular events and may experience functional and neurocognitive decline [45, 46], which, in our clinical experience, could affect psychosocial and mental health. Interestingly, while previous studies suggested a relation between sex and mental health, with females with FD reporting better psychological health [45, 47], this was not confirmed in the mental HRQOL model. An association between cardiac EF ≤ 50% and mental HRQOL is hard to explain. However, a moderately reduced EF (40–50%) seems unlikely to negatively affect mental health.

Since we started collecting study data right as the COVID-19 pandemic hit Norway, we saw the need to consider the pandemic’s impact on the SF-36 results obtained in 2020. In the present study, SF-36 data collected before the pandemic (baseline) were compared with SF-36 data collected during the pandemic. There were no significant differences (paired t-tests) between baseline and 2020 data (*n* = 36; PCS, *p* = 0.642 and MCS, *p* = 0.454). Based on this comparison, a general impact of COVID-19 on physical or mental HRQOL seems less likely.

This retrospective study integrates the strengths of an FD program with yearly clinical follow-up and systematically collected long-term medical data, providing us with the opportunity to study the same sample over time and to determine the clinical significance of medical complications on HRQOL. Considering that 100% of males in our study received ERT or chaperone treatment, it is plausible that such treatment may have haltered disease progression and hence reduced the potential negative relationships of kidney and cardiovascular diseases on HRQOL.

## Limitations

FD is a rare disease, and it is therefore challenging to obtain a large sample size, limiting the number of variables that can be included when modelling outcomes. One limitation is that disease severity was not evaluated in a structured manner (e.g., using the Mainz severity score index or the Fabry disease severity scoring system—DS3 [48, 49]) at each follow-up. Another limitation is that the cohort might not be representative of all individuals with FD in Norway. In addition, the 7–13 year timeframe is a long period and the data from baseline may be more representative of a younger Fabry population, while the data collected up to the 13-year HRQOL assessments may be more representative of older individuals.

Despite these potential limitations, the study sheds light on the influence of sex, age, and FD-related medical complications on HRQOL. This information may be used for informing health professionals, individuals with FD and their families, and in the development of health care services for individuals with FD.

## Conclusions

This retrospective longitudinal study is the first to describe the course, demographic and medical predictors of longitudinal HRQOL in adults with FD. The results suggest that HRQOL remains relatively stable over a period of 13 years. Age above 47 years, male sex, small fibre neuropathy, decreased renal function and a history of cerebrovascular events are related to lower physical HRQOL outcomes. Small fibre neuropathy did have a negative relationship on both physical and mental HRQOL trajectories. In addition, those with a cerebrovascular event reported decreased physical and mental HRQOL. Although the relationship between medical complications in FD and HRQOL remains complex, early identification of declining HRQOL may reveal opportunities to improve outcomes and quality of life. These findings have clinical implications (e.g., co-management of care delivery) and provide indicators (e.g., medical parameters, lab values) that can guide interventions to improve physical and mental health in individuals with FD. A deeper knowledge of how medical parameters are inter-related and how they associate with self-reported HRQOL might help us reach more well-founded treatment decisions in vulnerable patients. Insights obtained from studies of HRQOL in FD should guide clinicians from various disciplines to understand the patient’s needs early after diagnosis and allow for an individualized plan for future health care.

## Supplementary Information

**Additional file 1: Bar chart 1.** The X-axis shows the year of follow-ups, including the baseline (first visit). The Y-axis shows the frequency of SF-36 data included or not included in the database for longitudinal data. Total number of observations are indicated on the bars.

**Additional file 2: Figure S1A.** SF-36 domain scores for males with FD at follow-ups compared to the Norwegian male population.

**Additional file 3: Figure S1B.** SF-36 domain scores for females with FD at follow-ups compared to the Norwegian female population.

**Additional file 4: Figure S2.** Mental Component Summary Scores for 30 participants with FD between baseline and 3-5 year follow-ups.

**Additional file 5: Table S1.** Model fit for PCS and MCS trajectories over time.

## Data Availability

Norwegian ethical and legal restrictions prevent us from uploading data to public repositories or including the full dataset as Supplementary Material. Norwegian Fabry patients belong to a relatively small group, and very little personal data are needed to indirectly identify individual study participants. We have been in dialogue with the Data Protection Authority of Oslo University Hospital in this matter. Access to a limited version of the dataset containing selected variables may be made available on request.

## Abbreviations

FD: Fabry disease
HRQOL: Health-related quality of life
SF-36: The 36-item Short-Form Health Survey
PCS: Physical composite score
MCS: Mental composite score
PF: Physical Functioning
RP: Role-Physical
BP: Bodily Pain
GH: General Health
VT: Vitality
SF: Social Functioning
RE: Role-Emotional
MH: Mental Health
α-Gal-A: Enzyme α-galactosidase A
ERT: Enzyme replacement therapy
GFR: Glomerular filtration rate
mGFR: Measured glomerular filtration rate
ICD: Implantable cardioverter-defibrillator
EF: Cardiac ejection fraction

## References

[1] Zarate YA, Hopkin RJ (2008). Fabry's disease. Lancet.

[2] Mehta A, Ricci R, Widmer U, Dehout F, de Lorenzo AG, Kampmann C (2004). Fabry disease defined: baseline clinical manifestations of 366 patients in the Fabry Outcome Survey. Eur J Clin Invest.

[3] Hagege A, Reant P, Habib G, Damy T, Barone-Rochette G, Soulat G (2019). Fabry disease in cardiology practice: literature review and expert point of view. Arch Cardiovasc Dis.

[4] Schiffmann R, Hughes DA, Linthorst GE, Ortiz A, Svarstad E, Warnock DG (2017). Screening, diagnosis, and management of patients with Fabry disease: conclusions from a kidney disease: Improving Global Outcomes (KDIGO) Controversies Conference. Kidney Int.

[5] Beck M, Hughes D, Kampmann C, Larroque S, Mehta A, Pintos-Morell G (2015). Long-term effectiveness of agalsidase alfa enzyme replacement in Fabry disease: a Fabry Outcome Survey analysis. Mol Genet Metab Rep.

[6] Sims K, Politei J, Banikazemi M, Lee P (2009). Stroke in Fabry disease frequently occurs before diagnosis and in the absence of other clinical events: natural history data from the Fabry Registry. Stroke.

[7] Aguiar P, Azevedo O, Pinto R, Marino J, Baker R, Cardoso C (2017). New biomarkers defining a novel early stage of Fabry nephropathy: a diagnostic test study. Mol Genet Metab.

[8] Uceyler N, Magg B, Thomas P, Wiedmann S, Heuschmann P, Sommer C (2014). A comprehensive Fabry-related pain questionnaire for adult patients. Pain.

[9] Lidove O, Zeller V, Chicheportiche V, Meyssonnier V, Sene T, Godot S (2016). Musculoskeletal manifestations of Fabry disease: a retrospective study. Jt Bone Spine.

[10] Wanner C, Arad M, Baron R, Burlina A, Elliott PM, Feldt-Rasmussen U (2018). European expert consensus statement on therapeutic goals in Fabry disease. Mol Genet Metab.

[11] Arends M, Korver S, Hughes DA, Mehta A, Hollak CEM, Biegstraaten M (2018). Phenotype, disease severity and pain are major determinants of quality of life in Fabry disease: results from a large multicenter cohort study. J Inherit Metab Dis.

[12] Hughes DA, Nicholls K, Shankar SP, Sunder-Plassmann G, Koeller D, Nedd K (2017). Oral pharmacological chaperone migalastat compared with enzyme replacement therapy in Fabry disease: 18-month results from the randomised phase III ATTRACT study. J Med Genet.

[13] Spada M, Baron R, Elliott PM, Falissard B, Hilz MJ, Monserrat L (2019). The effect of enzyme replacement therapy on clinical outcomes in paediatric patients with Fabry disease—a systematic literature review by a European panel of experts. Mol Genet Metab.

[14] Meikle PJ, Hopwood JJ, Clague AE, Carey WF (1999). Prevalence of lysosomal storage disorders. JAMA.

[15] Deegan PB, Baehner AF, Barba Romero MA, Hughes DA, Kampmann C, Beck M (2006). Natural history of Fabry disease in females in the Fabry Outcome Survey. J Med Genet.

[16] Deegan PB, Bahner F, Barba M, Hughes DA, Beck M, Mehta A, Beck M, Sunder-Plassmann G (2006). Fabry disease in females: clinical characteristics and effects of enzyme replacement therapy. Fabry disease: perspectives from 5 years of FOS.

[17] Ivleva A, Weith E, Mehta A, Hughes DA (2018). The influence of patient-reported joint manifestations on quality of life in Fabry patients. JIMD Rep.

[18] Laney DA, Gruskin DJ, Fernhoff PM, Cubells JF, Ousley OY, Hipp H (2010). Social-adaptive and psychological functioning of patients affected by Fabry disease. J Inherit Metab Dis.

[19] Guffon N, Fouilhoux A (2004). Clinical benefit in Fabry patients given enzyme replacement therapy—a case series. J Inherit Metab Dis.

[20] Wagner M, Kramer J, Blohm E, Vergho D, Weidemann F, Breunig F (2014). Kidney function as an underestimated factor for reduced health related quality of life in patients with Fabry disease. BMC Nephrol.

[21] Wilcox WR, Oliveira JP, Hopkin RJ, Ortiz A, Banikazemi M, Feldt-Rasmussen U (2008). Females with Fabry disease frequently have major organ involvement: lessons from the Fabry Registry. Mol Genet Metab.

[22] Pascoal C, Brasil S, Francisco R, Marques-da-Silva D, Rafalko A, Jaeken J (2018). Patient and observer reported outcome measures to evaluate health-related quality of life in inherited metabolic diseases: a scoping review. Orphanet J Rare Dis.

[23] Arends M, Hollak CE, Biegstraaten M (2015). Quality of life in patients with Fabry disease: a systematic review of the literature. Orphanet J Rare Dis.

[24] Wang RY, Lelis A, Mirocha J, Wilcox WR (2007). Heterozygous Fabry women are not just carriers, but have a significant burden of disease and impaired quality of life. Genet Med.

[25] Street NJ, Yi MS, Bailey LA, Hopkin RJ (2006). Comparison of health-related quality of life between heterozygous women with Fabry disease, a healthy control population, and patients with other chronic disease. Genet Med.

[26] Rosa Neto NS, Bento JCB, Pereira RMR (2020). Depression, sleep disturbances, pain, disability and quality of LIFE in Brazilian Fabry disease patients. Mol Genet Metab Rep.

[27] Gold KF, Pastores GM, Botteman MF, Yeh JM, Sweeney S, Aliski W (2002). Quality of life of patients with Fabry disease. Qual Life Res.

[28] Hoffmann B, Schwarz M, Mehta A, Keshav S, Fabry Outcome Survey European I (2007). Gastrointestinal symptoms in 342 patients with Fabry disease: prevalence and response to enzyme replacement therapy. Clin Gastroenterol Hepatol.

[29] Uceyler N, Ganendiran S, Kramer D, Sommer C (2014). Characterization of pain in fabry disease. Clin J Pain.

[30] Burlina AP, Sims KB, Politei JM, Bennett GJ, Baron R, Sommer C (2011). Early diagnosis of peripheral nervous system involvement in Fabry disease and treatment of neuropathic pain: the report of an expert panel. BMC Neurol.

[31] Watt T, Burlina AP, Cazzorla C, Schonfeld D, Banikazemi M, Hopkin RJ (2010). Agalsidase beta treatment is associated with improved quality of life in patients with Fabry disease: findings from the Fabry Registry. Genet Med.

[32] Concolino D, Amico L, Cappellini MD, Cassinerio E, Conti M, Donati MA (2017). Home infusion program with enzyme replacement therapy for Fabry disease: the experience of a large Italian collaborative group. Mol Genet Metab Rep.

[33] Germain DP, Elliott PM, Falissard B, Fomin VV, Hilz MJ, Jovanovic A (2019). The effect of enzyme replacement therapy on clinical outcomes in male patients with Fabry disease: a systematic literature review by a European panel of experts. Mol Genet Metab Rep..

[34] Hoffmann B, de Lorenzo AG, Mehta A, Beck M, Widmer U, Ricci R (2005). Effects of enzyme replacement therapy on pain and health related quality of life in patients with Fabry disease: data from FOS (Fabry Outcome Survey). J Med Genet.

[35] Rombach SM, Hollak CE, Linthorst GE, Dijkgraaf MG (2013). Cost-effectiveness of enzyme replacement therapy for Fabry disease. Orphanet J Rare Dis.

[36] Orstavik K, Norheim I, Jorum E (2006). Pain and small-fiber neuropathy in patients with hypothyroidism. Neurology.

[37] Rombach SM, Baas MC, ten Berge IJ, Krediet RT, Bemelman FJ, Hollak CE (2010). The value of estimated GFR in comparison to measured GFR for the assessment of renal function in adult patients with Fabry disease. Nephrol Dial Transplant.

[38] Ware JE, Snow KK, Kosinski M, Gandek B (1993). SF-36 health survey: Manual and interpretation guide.

[39] Hopman WM, Berger C, Joseph L, Zhou W, Prior JC, Towheed T (2014). Prospectively measured 10-year changes in health-related quality of life and comparison with cross-sectional estimates in a population-based cohort of adult women and men. Qual Life Res.

[40] Garratt AM, Stavem K (2017). Measurement properties and normative data for the Norwegian SF-36: results from a general population survey. Health Qual Life Outcomes.

[41] Jacobsen EL, Bye A, Aass N, Fossa SD, Grotmol KS, Kaasa S (2018). Norwegian reference values for the Short-Form Health Survey 36: development over time. Qual Life Res.

[42] Smid BE, Rombach SM, Aerts JM, Kuiper S, Mirzaian M, Overkleeft HS (2011). Consequences of a global enzyme shortage of agalsidase beta in adult Dutch Fabry patients. Orphanet J Rare Dis.

[43] Biegstraaten M, Binder A, Maag R, Hollak CEM, Baron R, van Schaik IN (2011). The relation between small nerve fibre function, age, disease severity and pain in Fabry disease. Eur J Pain.

[44] Politei JM, Bouhassira D, Germain DP, Goizet C, Guerrero-Sola A, Hilz MJ (2016). Pain in Fabry disease: practical recommendations for diagnosis and treatment. CNS Neurosci Ther.

[45] Sigmundsdottir L, Tchan MC, Knopman AA, Menzies GC, Batchelor J, Sillence DO (2014). Cognitive and psychological functioning in Fabry disease. Arch Clin Neuropsychol.

[46] Korver S, Geurtsen GJ, Hollak CEM, van Schaik IN, Longo MGF, Lima MR (2019). Predictors of objective cognitive impairment and subjective cognitive complaints in patients with Fabry disease. Sci Rep.

[47] Cole AL, Lee PJ, Hughes DA, Deegan PB, Waldek S, Lachmann RH (2007). Depression in adults with Fabry disease: a common and under-diagnosed problem. J Inherit Metab Dis.

[48] Giannini EH, Mehta AB, Hilz MJ, Beck M, Bichet DG, Brady RO (2010). A validated disease severity scoring system for Fabry disease. Mol Genet Metab.

[49] Whybra C, Kampmann C, Krummenauer F, Ries M, Mengel E, Miebach E (2004). The Mainz Severity Score Index: a new instrument for quantifying the Anderson-Fabry disease phenotype, and the response of patients to enzyme replacement therapy. Clin Genet.

