# Supplementary material for: The impact of demographic and clinical characteristics on the trajectories of health-related quality of life among patients with Fabry disease
Source: Orphanet J Rare Dis. 2021 Oct 12;16:427. doi: 10.1186/s13023-021-02066-y (PMC8506470; doi:10.1186/s13023-021-02066-y)
Supplement: Supplementary file 1 — Additional file 1: Bar chart 1. The X-axis shows the year of follow-ups, including the baseline (first visit). The Y-axis shows the frequency of SF-36 data included or not included in the database for longitudinal data. Total number of observations are indicated on the bars. [file 13023_2021_2066_MOESM1_ESM.docx]

**Bar Chart 1.** The X-axis shows the year of follow-ups, including the baseline (first visit). The Y-axis shows the frequency of SF-36 data included or not included in the database for longitudinal data. Total number of observations are indicated on the bars.
