# Supplementary material for: The impact of demographic and clinical characteristics on the trajectories of health-related quality of life among patients with Fabry disease
Source: Orphanet J Rare Dis. 2021 Oct 12;16:427. doi: 10.1186/s13023-021-02066-y (PMC8506470; doi:10.1186/s13023-021-02066-y)
Supplement: Supplementary file 3 — Additional file 3: Figure S1B. SF-36 domain scores for females with FD at follow-ups compared to the Norwegian female population. [file 13023_2021_2066_MOESM3_ESM.docx]

**Figure S1B.** SF-36 domain scores for females with FD at follow-ups compared to the Norwegian female population
