# Supplementary material for: The impact of demographic and clinical characteristics on the trajectories of health-related quality of life among patients with Fabry disease
Source: Orphanet J Rare Dis. 2021 Oct 12;16:427. doi: 10.1186/s13023-021-02066-y (PMC8506470; doi:10.1186/s13023-021-02066-y)

**Figure S2**.

Mental Component Summary Scores for 30 participants with FD between baseline and 3-5 year follow-ups.


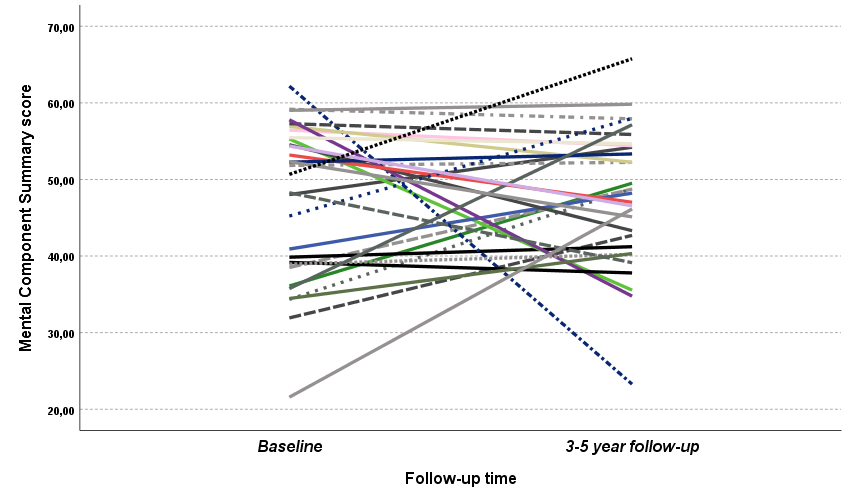

Supplement: Supplementary file 4 — Additional file 4: Figure S2. Mental Component Summary Scores for 30 participants with FD between baseline and 3-5 year follow-ups. [file 13023_2021_2066_MOESM4_ESM.docx]
