# Supplementary material for: The impact of demographic and clinical characteristics on the trajectories of health-related quality of life among patients with Fabry disease
Source: Orphanet J Rare Dis. 2021 Oct 12;16:427. doi: 10.1186/s13023-021-02066-y (PMC8506470; doi:10.1186/s13023-021-02066-y)
Supplement: Supplementary file 5 — Additional file 5: Table S1. Model fit for PCS and MCS trajectories over time. [file 13023_2021_2066_MOESM5_ESM.docx]

Table S1. Model fit for PCS and MCS trajectories over time

| **Model Physical Component Summary (PCS)** | **-2 Log Likelihood** |
| --- | --- |
| Linear | 706.77 |
| Quadratic | 711.43 |
| Cubic | 716.13 |
| **Model Mental Component Summary (MCS)** |  |
| Linear | 653.79 |
| Quadratic | 658.33 |
| Cubic | 662.97 |

*Note*. The Chi-square value for significant difference (*p* = .05) is > 3.84 drop from the previous model. Lower -2 Log Likelihood is better.

The quadratic and cubic effects did not improve model-fit based on -2 log Likelihood (-2LL), Akaike information criterion and Bayesian information criterion. A linear shape trajectory emerged as the best fitting to the data in the PCS and MCS analyses over time.
